# Partnerships in global health and collaborative governance: lessons learnt from the Division of Tropical and Humanitarian Medicine at the Geneva University Hospitals

**DOI:** 10.1186/s12992-016-0156-x

**Published:** 2016-04-29

**Authors:** David Beran, Sigiriya Aebischer Perone, Gabriel Alcoba, Alexandre Bischoff, Claire-Lise Bussien, Gilles Eperon, Olivier Hagon, Olivia Heller, Frédérique Jacquerioz Bausch, Nicolas Perone, Thomas Vogel, François Chappuis

**Affiliations:** Division of Tropical and Humanitarian Medicine, Geneva University Hospitals, Geneva, Switzerland; Faculty of Medicine, University of Geneva, Geneva, Switzerland

**Keywords:** Partnerships, Collaborations, Hospitals, Global health, Governance

## Abstract

**Background:**

In 2007 the “Crisp Report” on international partnerships increased interest in Northern countries on the way their links with Southern partners operated. Since its establishment in 2007 the Division of Tropical and Humanitarian Medicine at the Geneva University Hospitals has developed a variety of partnerships. Frameworks to assess these partnerships are needed and recent attention in the field of public management on collaborative governance may provide a useful approach for analyzing international collaborations.

**Methods:**

Projects of the Division of Tropical and Humanitarian Medicine were analyzed by collaborators within the Division using the model proposed by Emerson and colleagues for collaborative governance, which comprises different components that assess the collaborative process.

**Results:**

International projects within the Division of Tropical and Humanitarian Medicine can be divided into four categories: Human resource development; Humanitarian response; Neglected Tropical Diseases and Noncommunicable diseases. For each of these projects there was a clear leader from the Division of Tropical and Humanitarian Medicine as well as a local counterpart. These individuals were seen as leaders both due to their role in establishing the collaboration as well as their technical expertise. Across these projects the actual partners vary greatly. This diversity means a wide range of contributions to the collaboration, but also complexity in managing different interests. A common definition of the collaborative aims in each of the projects is both a formal and informal process. Legal, financial and administrative aspects of the collaboration are the formal elements. These can be a challenge based on different administrative requirements. Friendship is part of the informal aspects and helps contribute to a relationship that is not exclusively professional.

**Conclusion:**

Using collaborative governance allows the complexity of managing partnerships to be presented. The framework used highlights the process of establishing collaborations, which is an element often negated by other more traditional models used in international partnerships. Applying the framework to the projects of the Division of Tropical and Humanitarian Medicine highlights the importance of shared values and interests, credibility of partners, formal and informal methods of management as well as friendship.

## Background

In 2007 the United Kingdom (UK) assessed the contribution of partnerships to improving global health and highlighted the responsibility of “Northern” based institutions [1]. This report emphasized both the challenges and opportunities that these international collaborations represent for partners in both the global North and South. In classifying international collaborations, Gaillard [2] divides these into: technical assistance; overseas training; institution building; institutional twinning and collaborative research. This is very similar to areas of international collaboration involving hospitals, which usually include activities around the themes of: training of health professionals; provision of actual health care; projects in improving management of facilities; use of innovative technologies and research [3]. The Division of Tropical and Humanitarian Medicine (DTHM) at the Geneva University Hospitals (HUG) in Switzerland is a rare example of a division within a public teaching hospital dedicated to improving health globally. Its activities can be divided into those benefitting the population of Geneva through its travel medicine clinics as well as those benefitting the wider global population through projects and research. Since its establishment in 2007 it has developed a variety of partnerships within the HUG and outside, in Geneva, Switzerland and throughout the world to address its core mission of “developing partnerships with local and international organizations, favoring an interdisciplinary and interactive approach, to enable improving access to health taking advantage of the skills available at the HUG and engaging them in international activities.” [4]. This builds on both the clinical expertise present within one of the leading academic Swiss hospitals, as well as Geneva being home to humanitarian principles and many international organizations and NGOs involved in global health.

Different terms have been used to describe partnerships, such as twinning, links and collaborations. For example in the UK links are characterized by “long-term mutually beneficial partnerships” which allow for this benefit to be both for the partners in the North and South in terms of knowledge and skills [5]. The concept of twinning also includes this element of the outcomes being beneficial to all partners [6]. Googins and Rochlin [7] argue that partnerships are an opportunity to build something between the partners that they would not be able to do alone. Within these three definitions a common term exists that of “mutually”. This term is extremely important as from a historical context international projects were seen to primarily benefit recipients [8]. Parry and Percy [9] highlight that the mutual benefits of collaborative projects between “North and South” are personal, awareness of different cultures, creativity, additional experience from a different setting in the area of expertise (for individuals and institution), motivating factor for attracting and retaining staff, and career development.

In the literature there has been much discussion of these health partnerships in terms of benefits for both partners and the challenges they may encounter, issues of trust, the time and resources needed to develop these partnerships, capacity of partners in developing countries, issues of governance, agenda and that the definition of priorities is often driven by Northern partners, role of each partner, asymmetry of relationships and how to document success of joint work [2, 5, 6, 10–14]. Frameworks to assess these partnerships are needed and recent attention in the field of public management on collaborative governance may provide a useful approach for analyzing international collaborations.

Collaborative governance is focused on bringing together a variety of stakeholders such as governments, the private sector and civil society and how these different sectors can effectively collaborate despite their different backgrounds, modes of operation and interests [15, 16]. Within this governance is defined as “a set of coordinating and monitoring activities” which allow for an effective collaboration or partnership [17]. In the context of collaborative governance the concept of governance focuses on how this works across a network of different actors, both formal and informal, and how this can help or hinder the progress of joint activities [15]. Zadek [18] adds to these definitions in that collaborative governance establishes the institutional arrangements and rules that allow for multi-stakeholder collaboration. This is both in terms of how the collaboration will work and the perception of the role of each partner. For the purpose of this article the definition of collaborative governance that will be used is the one proposed by Emerson et al. [17] “as the processes and structures of public policy decision making and management that engage people constructively across the boundaries of public agencies, levels of government, and/or the public, private and civic spheres in order to carry out a public purpose that could not otherwise be accomplished.” The authors of this definition add that this can also be applied to the issue of “multi-partner” governance with any mix of institutions included.

The aim of this article is to apply concepts from collaborative governance to the subject of international health partnerships and use the example of the activities from the DTHM to highlight the lessons learnt which might be useful for the analysis of global health partnerships.

## Methods

One of the frameworks used in the context of collaborative governance is the model proposed by Emerson et al. [17], which proposes to look at a variety of components that help assess the collaborative process. For the purpose of this article certain selected components of this framework are chosen (Table 1). The approach chosen was to focus on the process elements, as these are often an overlooked aspect of collaborations. Also traditional frameworks for presenting international collaborations [7, 10, 13, 19–21] in health have overlapping factors as included in Emerson et al.’s [17] model (Table 2). For example 5 out of the 11 key principles of the Swiss Commission for Research Partnerships with Developing Countries (KFPE) [20] are present in this framework. The missing elements focus more on the outcomes of partnerships than the process. Lowndes and Skelcher [21] look at the process of collaboration as 4 phases, Pre-partnership collaboration, Partnership creation and consolidation, Partnership program delivery and Partnership termination and succession. These two first stages fit into Emerson et al.’s [17] model as the process of establishing the partnership in this study. Table 2 shows how the model used provides a comprehensive overview of issues addressed in these other frameworks.

**Table 1:** Elements from Emerson et al.’s [17] model of collaborative governance

Drivers of the collaboration	Members of the collaboration	Principled engagement	Shared motivation	Capacity for joint collaboration
- Leadership - Consequential incentives - Interdependence - Uncertainty	- Skills - Strengths - Contribution to the project	- Discovery - Definition - Deliberation - Determination	- Mutual trust - Mutual understanding - Internal legitimacy - Shared commitment	- Procedural and institutional arrangements - Leadership - Knowledge - Resources

**Table 2:** Comparison of different frameworks for international collaboration with Emerson et al.’s [17] model of collaborative governance focusing on the development and implementation of the collaboration

	Drivers of the collaboration	Members of the collaboration	Principled engagement	Shared motivation	Capacity for joint collaboration
Dowling et al. [19]	- Agreement on need for collaboration		- Agreement on purpose of collaboration	- Engagement and commitment - Trust, reciprocity and respect	- Favorable environment - Accountability procedures - Leadership and management
Googlins and Rochlin [7]	- Obtaining commitment from leadership		- Defining clear goals		- Frequent communication - Allocating human resources for specific tasks - Sharing of resources
Huxham et al. [13]	- Working relationships - Need for multiple partners	- Members partaking in the collaboration			- Working relationships - Governance and responsibilities - Representatives of collaboration
KFPE [20]			- Setting the agenda together	- Interaction with stakeholders - Promoting mutual learning	- Clarifying responsibilities - Sharing data and networks
Lasker et al. [10]	- Leadership - Management			- Partner participation - Partner relationships	- Staff support - Sufficient resources - Management - Communication - Governance - Partnership structure
Lowndes and Skelcher [21]	- Pre-partnership collaboration		- Partnership creation and consolidation	- Pre-partnership collaboration	- Partnership creation and consolidation

One of the elements included in Emerson et al.’s [17] model is the “Drivers” of the collaboration. Included in these is “leadership”, which is the presence of an individual who is seen as a leader. This role as a leader may be due to their position in one of the partner organizations, their technical expertise (a leader in the field) or their role in the creation of the collaboration. The next driver is termed “consequential incentives”. These are the factors in both the internal and external environments that drive the collaboration. Included in these are:

- Problems
- Resource needs
- Interests
- Opportunities (e.g. availability of a grant)

This allows for the collaboration to be presented in a way to others that allows it to be seen as something important and enables the different parties to engage with each other. “Interdependence” is needed to initiate the collaboration, as each member in the partnership is unable to undertake the specific activity without the other participating. The final driver is that of “uncertainty” this is the lack of a solution that each partner may have individually calling into play the need for collaboration to identify ways of addressing this. These are the elements that are needed to “drive” partners to collaborate.

The next elements focus on how the collaboration is shaped and developed. The first of these are the people involved in the collaboration with their skills and strengths and how these will contribute to the project. Emerson et al. [17] refer to this component as “principled engagement”. Within this component one of the most important factors is the actual members of the collaboration. The importance of this is to get the right people from different perspectives (technical, political, etc.) to collaborate and bring their different skills to the benefit of the project. Principled engagement describes 4 processes: discovery, definition, deliberation, and determination. These elements help advance the development of the collaboration in terms of purpose, understanding of the problem as well as the proposed course of action to address this. Discovery is defined as the realization of “shared interests, concerns, and values”. The next process, definition, looks at the efforts that aim to come to a common “definition” of purpose and objectives of the collaboration. Communication within the project falls within the deliberation process and how different interests and perspectives are discussed and agreed upon for the benefit of the project. The last element is how joint decisions, determinations, are made including different types of determinations that allow the collaboration to progress. These include procedural decisions, those that enable the project to move forward (e.g. agendas, planning discussion groups or working groups) as well as substantive decisions that help with main milestones of the project (agreement on main objectives and final outputs).

Shared motivation is the next category included in Emerson et al.’s [17] framework and used in this analysis. This is composed of mutual trust, understanding, internal legitimacy, and commitment, which focus on the interpersonal and relational aspects of the collaboration process. Mutual trust is developed over time as the collaboration moves forward and each partner shows that they can be trusted. This helps develop the next element of mutual understanding. Mutual understanding refers to the partners in the collaboration understanding and respecting their colleagues’ views and positions. The next stage is internal legitimacy with the participants in the collaboration being seen as “trustworthy and credible” and that the shared interests creates a cycle of legitimizing and motivating the ongoing joint work. Shared commitment is the commitment to the overall process of the collaboration.

As detailed in Table 1 the fifth element describes how within a collaboration new capacities need to be developed to enable it to be successful and requires 4 elements: procedural and institutional arrangements, leadership, knowledge, and resources. These elements need to be present in sufficient amounts to ensure a successful collaboration. The first of these elements include a range of procedural mechanisms that are defined both within each organization and between organizations. Leadership is also included in capacity for collaboration in that a leader is needed for the different functions of the collaboration, e.g. representation, convener and/or facilitator. Knowledge is essential to the collaboration and needs to be shared with others involved in the collaboration as well being generated by the joint work. This knowledge also needs to be able to circulate within the collaboration and therefore mechanisms need to be put in place. Resources are both essential to the collaboration and a potential benefit of collaborations in that they are able to share and leverage new resources. Of course financial and other resources are necessary for each collaboration and these can be “leveraged and redistributed” from each member of the collaboration.

During the annual review meeting of the DTHM held in March 2015 all projects from 2014 (completed and ongoing) as well as planned projects for 2015 were presented by the project lead. AB, CLB, DB, FC, GA, GE, NP, OH, OHE and TV were present at the meeting. Each project was discussed in length in terms of various factors (e.g. challenges, new perspectives, results and next steps) as well as focusing on the actual partners and partnerships that formed part of the project. Based on the presentations and report from this meeting DB presented an initial analysis of completed and ongoing projects from 2014 using the framework proposed by Emerson et al. [17] for discussion to the other authors. Further elements of the analysis were added by each of the co-authors based on their own projects as well as their understanding of their colleagues’ projects. This was an iterative process and any discordance was addressed by DB, either through one-on-one discussions, discussions during DTHM weekly meetings or in the text used as a basis for this paper. This of course only provides the “Northern” perspective on these partnerships and how they apply to the framework used.

## Results

**Fig. 1:**
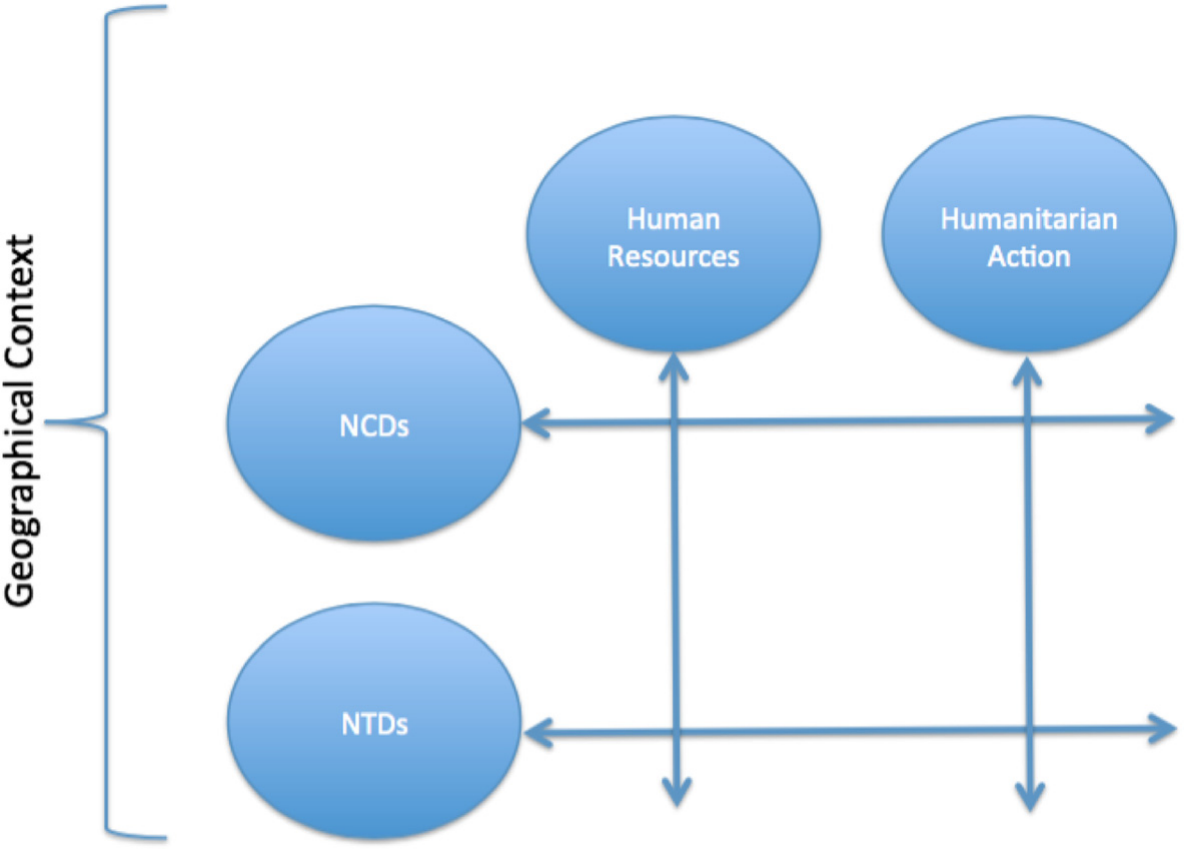
Activity matrix of the DTHM

The international projects, both development and research, within the DTHM can be divided into four categories: Human resource and institutional development; Humanitarian response; Neglected Tropical Diseases (NTD) and Noncommunicable diseases (NCD). The way these different themes are organized can be viewed as both horizontal and vertical approaches to these different elements, with for example important components of human resource development or the humanitarian response also including aspects of NCDs. This is presented in Fig. 1. The focus on these four elements allows the DTHM to concentrate its resources and expertise as well as clearly establish potential areas of collaboration. These projects are also concentrated in certain countries aligning geographical, thematic and methodological approaches. A summary of these projects is presented in Table 3 a and b.

**Table 3:** Summary description of each DTHM project

	Country	Brief description	Partners	Funder
Human Resources and Institutional Development	Kyrgyzstan	Pre graduate, post-graduate and continuous medical education reforms	Local NGO, Ministry of Health, Higher Education institutions	SDC
	Togo	Continuing training of nurses	National nurses association	HUG special humanitarian fund
	Tanzania	Development of nursing school	Local health authorities and hospital, Faith Based Organizations	International Office for Solidarity of the Canton of Geneva
	Bosnia-Herzegovina	Primary care reforms	Local NGO, Local health authorities, Ministry of Health, healthcare workers	SDC
Humanitarian Action	Various	Provision of human and technical resources during different humanitarian emergencies	NGOs (e.g. MSF), SDC, local organizations and authorities	SDC and other sources
	Liberia and Guinea	Assistance during Ebola crisis	Other divisions at HUG, NGOs (e.g. MSF, WHO), local partners from private and public sector and Ministry of Health	SDC
	Jordan	Development of ambulance services	Local partners and authorities, private sector in Switzerland	State Secretariat for Economic Affairs
NTD related projects	Nepal	Long-term research and exchange projects	Local research institution and local health authorities	Various
	Various	Research and operational research, improving the clinical management of NTDs and workshops and trainings	Research institutions (North and South), MSF, local healthcare workers and health institutions	Various
NCD related projects	Bosnia-Herzegovina	Improving the management of mental health	Local health authorities, Ministry of Health, healthcare workers	SDC and different Swiss Cantons
	Mali	Improving the management of diabetes	Local NGO, Ministry of Health	Various
	Zanzibar	Development of a national NCD strategy	Ministry of Health, WHO	WHO
	Peru	Assessment of management of diabetes and hypertension	Local research institution, Local health authorities, Ministry of Health, WHO	WHO

### Human resource and institutional development projects

Since 2007 the DTHM has been involved in the medical education reform in Kyrgyzstan in the form of technical support to medical faculties and the Ministry of Health. In Switzerland, this includes collaboration with the University of Geneva Faculty of Medicine. Initially focused on Pre-graduate medical education, since 2013 this project has also included Postgraduate and Continuing Medical Education. In 2014 a new phase of the project was initiated with more active involvement of the DTHM as well as developing a partnership with a local NGO for implementation. This project is financed by the Swiss Agency for Development and Cooperation (SDC).

A specific area of expertise of the DTHM in terms of Human Resource Development is nursing. A training program in Togo in collaboration with the Togolese Association of Nurses has focused on Continuing training for nurses in certain areas of expertise of the HUG, for example ethics, diabetes and management. Staff from the DTHM and other colleagues from the HUG partnered with Togolese colleagues to design and deliver these training courses based on topics chosen locally. What is also interesting with this project is that it is supported by a special humanitarian fund established within the HUG that uses income generated by the private consultations of HUG specialists to fund international projects.

Another nursing project has been the development of a nursing school in Tanzania. This has enabled the establishment of a 3-year diploma course in nursing with funding from the International Office for Solidarity of the Canton of Geneva. This support was for infrastructure, administration and development of Training of Trainers programs, including teaching activities for students.

For 20 years the DTHM and the Faculty of Medicine of the University of Geneva have been involved in supporting the primary care reform in Bosnia-Herzegovina. Currently a 9-year project focusing on strengthening nursing based on three components, namely, community nursing, basic nurse training and continuous professional development. The DTHM brings its technical expertise and capacity building, coordination and quality control to local institutions to this project, which is also funded by the SDC. This project benefits from the close collaboration and trust relationship created over years with the Bosnian authorities and institutions. A consortium of three partners implements the project: a local NGO (Fondacija Fami) making the link with the local health authorities and institutions at national and regional level, HUG and the Institute of Nursing Sciences of Basel University. The project facilitates partnership with Bosnian health authorities to allow nurses taking up responsibility for the performance of their profession and to recognize nurses as an important resource for improved health in line with European good practices, with adapted job descriptions for community nurses including an expanded scope of practice requiring educational, operational, organizational and material changes.

### Humanitarian action

Another area of know-how of the DTHM is humanitarian action in directly involving its collaborators during complex emergencies (e.g. earthquake in Haiti, typhoon in the Philippines), enabling the temporary recruitment of other HUG collaborators with governmental (e.g. humanitarian aid of the SDC) or non-governmental (e.g. Médecins sans Frontières: MSF) organizations, providing medical expertise in NTD and NCD in humanitarian crisis settings, e.g. sleeping sickness MSF control program in Northeast Democratic Republic of Congo, development of guidelines on NCD in complex emergencies and teaching pre-graduate and post-graduate students, the latter at the Geneva Centre for Education and Research in Humanitarian Action based at the University of Geneva and the Graduate Institute.

In collaboration with other divisions of the HUG, the DTHM has been actively involved in the recent Ebola crisis. It coordinated the implementation of local production of alcohol-based hand rub solution (ABHRS) in Liberia and Guinea and directly provided care to returning expatriates or travelers with a history of exposure to body fluids of an infected patient and/or clinical symptoms consistent with Ebola. It also played an important coordinating role in shaping the response of the Swiss authorities to this crisis both abroad and for Switzerland. This led the humanitarian aid branch of the SDC to support the HUG in various activities, such as sending collaborators to the field (Sierra Leone) to support MSF clinical activities and training Guinean and Liberian health workers at the HUG on infection prevention and control and the development of portable laboratories.

Related to the ongoing Middle East humanitarian crisis, the DTHM manages a project with the government in Jordan to develop their ambulance services. This project includes the purchase of vehicles, training and development of quality systems in collaboration with Jordanian partners as well as an ambulance manufacturer in Switzerland.

### Neglected tropical diseases related projects

NTDs represent a disease area where the DTHM can be viewed as an international leader in terms of research. Long-term collaborations in this area with the B.P. Koirala Institute of Health Sciences (BPKIHS) in Dharan, Nepal, have led to a variety of spin-off projects such as exchange programs of students (Geneva to Nepal) and medical doctors (Nepal to Geneva) or extension of the research partnerships to other medical fields. Research projects on NTDs and other neglected health problems in Nepal have mainly focused on visceral leishmaniasis and snakebites. The choice of the latter was based on the identified need by both partners that this is among the top ten killers in some villages in Nepal [22].

Other research and/or operational projects in the area of NTDs include visceral leishmaniasis (Kenya, Sudan, Uganda) and sleeping sickness (Democratic Republic of the Congo, South Sudan) in collaboration with MSF, improvement of diagnostic algorithms for individuals with neurological disorders, persistent fever or digestive symptoms in several NTD endemic countries (www.nidiag.org), epidemiological and diagnostic studies on strongyloidiasis in Bolivia, and Chagas disease in migrant populations in Geneva. This expertise also leads to the DTHM being part of different expert groups within the World Health Organization (WHO).

### Noncommunicable diseases related projects

In the area of NCDs the DTHM has been involved in a mental health project in Bosnia-Herzegovina since 2013. This project focuses on quality improvement, capacity building, occupational health, prevention and health promotion, continuing training of health professionals and decreasing stigma and discrimination of patients.

Similar to its involvement in NTDs, with NCDs the DTHM has developed its expertise in a variety of areas with regards to access to medicines and health systems for the management of NCDs and this has meant substantial involvement in a variety of projects and policy discussions, for example participating in technical expertise and working groups within the WHO. Some specific projects in this area have included a health systems assessment in Peru looking at barriers to care for diabetes and hypertension, development of a national NCD plan in Zanzibar and technical support to an NGO active in the area of diabetes in Mali. NCDs are also being dealt with transversally in that care for chronic conditions is a main topic in all nursing-related projects (Tanzania, Togo and Bosnia-Herzegovina).

## Analysis

In looking at these different projects described and the framework proposed by Emerson et al. [17] different lessons can be learnt from the experience of the DTHM as presented below and in Table 4.

**Table 4:** Presentation of DTHM’s activities using Emerson et al.’s [17] model of collaborative governance

Drivers of the collaboration	Members of the collaboration	Principled engagement	Shared motivation	Capacity for joint collaboration
- Clear leadership at DTHM and in partner institutions - Technical expertise of HUG staff and local partners - View of being experts outside of partnership - Mix and complementarity of skills between North and South partners - Addressing complex issues with no set recipe	- Variety and range of skills present within DTHM and colleagues, technical/academic as well as field experience - DTHM includes clinicians, nurses as well as public health specialists, with a breadth and depth of expertise - Partners included in these collaborations represent a range of institutions - Partners also have supplementary or complementary skills - Partnership is not always between two partners, might include many - DTHM information/expertise broker with other experts at HUG and University of Geneva	- Shared values and interests - Role of funders - Formal and informal procedures - Skill mix within DTHM - Experience in finding locally adapted solutions - Role of being active in different networks	- Relationships within project going beyond professional and including personal friendships - Trust - Membership to different expert networks leads to DTHM’s staff being seen as credible partners - Experience of DTHM and staff	- Administrative challenges - Challenges in managing projects in different and difficult contexts - Resources mainly from North - Complexity of partnerships increases with number of partners involved - Leadership: identified leaders of projects as well as being a technical leader in the area of interest - Communication tools

### Drivers of the collaboration

For each of these projects there was a clear leader/coordinator from the DTHM as well as a local counterpart. These individuals were seen as leaders both due to their role in establishing the collaboration as well as their technical expertise. With regards to the Tanzania project, the DTHM collaborator had both a coordinating and advisory role. The DTHM coordinator went to Mbozi three times a year and was in charge of the donor’s budget. Therefore the financial “leadership” in terms of the needed investments to be funded was made in Geneva. With local stakeholders, the nursing school, the hospital, the Ministry of Health and its department of human resources, as well as the church leaders, the DTHM project leader had an advisory role, and communicated with the nursing school principal via phone, SMS and email regularly.

In the case of the partnership with BPKIHS in Nepal, the triggering events were: the visit of the DTHM by the BPKIHS vice-Chancellor (at the time of the 1998 World Health Assembly) to assess the potential availability and motivation of the DTHM to launch a collaboration with his institute and a 4-week visit of a DTHM collaborator to the BPKIHS to identify research fields of common interest and collaborators with a similar degree of motivation. Two young doctors were identified during this initial visit, and have since then become leading experts in the fields of visceral leishmaniasis and snakebites in Nepal and abroad, and continue to lead research and advocacy projects with the same DTHM collaborator more than 15 years later. In the case of the partnership with MSF at headquarter level in Geneva, one member of the team is working 50 % at the DTHM and the other 50 % at MSF on tropical medicine projects relevant to both institutions.

At the start of the Ebola crisis, the availability of technical expertise within the DTHM and from its close collaborators at the HUG, strong partnerships with WHO and MSF, and pre-existing relations with the authorities in the affected countries (e.g. Liberia) placed the DTHM in a privileged position to obtain financial support and play a leading role in the Swiss Ebola response both nationally and in West Africa. This project is characterized by a co-leadership with the Division of infectious diseases and encompasses a broad range of activities from field implementation of the production of ABHRS in Liberia and Guinea to training opportunities for southern collaborators in Geneva and the development of portable laboratories. The need for involvement in this humanitarian crisis was obvious and overwhelming with specific requests from authorities in the affected countries, the need for rapid action, and the international outbreak response landscape and partners shaped the development of these distinct and multi-country activities.

With regards to “consequential incentives” the examples of NTDs and NCDs are interesting to look at. Both issues are clear public health problems with a variety of resources needed. These global problems do not have ready-made solutions, for example in terms of diagnosis and treatment for NTDs or delivery of care for NCDs. The main resource the DTHM provided in this was technical expertise and easier access to grants. For example, the first collaborative research project on visceral leishmaniasis in Nepal was funded by the HUG internal funding mechanism for international projects mentioned above and by a WHO grant obtained through a visit and discussion of the DTHM collaborator with the WHO leishmaniasis coordinator at the WHO headquarters, following a 8 km bus ride. Similar opportunities arose for NCDs where through discussions, meetings and participation in networks the DTHM was able to work together with partners in Peru on a health system assessment and in Zanzibar with the development of a national NCD plan. With these elements present DTHM was actively able to engage partners in countries to address these challenges in addition to more global partnerships with organizations such as MSF or the WHO.

Interdependence is an interesting element to look at for all projects as the DTHM and their in-country partners each brought their unique know-how to the partnership. In the case of the Tanzanian Nursing School project, it is very clear that without the initiative of local partners, the project would not have been able to be launched and achieve the accreditation of the nurse training according to the National accreditation board. However, this was highly dependent on the DTHM’s provision of human and financial resources. Another example in the area of NCDs was in Zanzibar where the DTHM contribution was a health systems and integrated view of how to address the challenge of NCDs with local partners adding their more practical and country specific experience.

The lack of a solution in all these cases resulted in the need for collaboration. The complexity of addressing medical education reform in Kyrgyzstan meant that local partners via the SDC required external technical support to help further these reforms not necessarily by the DTHM providing answers, but facilitating the process. In terms of both Humanitarian Action and NCDs here again the DTHM has been a facilitator to organizations such as MSF providing technical expertise or assisting in a process of developing a solution.

### Members of the collaboration

Across these projects the actual partners working with DTHM vary greatly from Ministries of Health, international organizations, NGOs (both local and international), medical and nursing faculties, universities, medical professionals and researchers as well local populations. Each of these partners brings certain skills, strengths and therefore has a different contribution to the project. The Nursing School Project in Mbozi, Tanzania is both a good and bad example about having different types of members involved in one project. Good, because many stakeholders are kept on board, that would otherwise lead to unhealthy rivalry, and bad, because bringing together so many different experts and expertise is a time consuming process.

Another challenging project is one in Bosnia and Herzegovina, a divided country with two entities. In this project the DTHM has to deal simultaneously with politicians, health staff, health policy makers, civil society, NGOs, Swiss management structures and Bosnian management structures, as well as different (including centralized or decentralized) financial and management mechanisms. The Jordan project is the only ongoing project within the DTHM where the private sector is directly involved. This adds to complexity of the project as well as challenges that are not traditionally encountered in development projects, such as dealing with contracts and other interactions with a business orientation that an organization focused on health and development projects is not used to deal with.

The collaboration between HUG/DTHM and the nursing association in Togo allows for more recognition for the nursing profession and allows it to have more influence at the level of health officials. In Geneva, this project allowed colleagues to be able to participate in a different type of project that they would usually not be involved with, with different colleagues and therefore strengthened the network within the HUG between different individuals.

### Principled engagement

Realizing “shared interests, concerns, and values” is an interesting process to look at specifically in the Nepal collaboration and the nursing project in Tanzania. In Tanzania, the “discovery phase” with its shared interests, comprised collaborators from government and Ministry of Health, a Faith-based organization, a district hospital and district health officials and Swiss experts. Thanks to prevailing PHC movement with the urgency to train so many nurses to staff every dispensary, these partners had a shared view.

In Togo it was not easy to deliver a nursing training course according to the needs and wishes of local partners. The training needed to take into account the different roles of nurses between contexts, their link with doctors and overall role and level of responsibility within the health system. Although the contexts in Geneva and Togo with regards to these issues are different the shared interest and view of the role of nurses enabled this project to address identified challenges.

Elaborating common collaborative aims in each of the DTHM’s projects is a process both formal and informal. The formal aspects are the terms of reference and various agreements that define the purpose and objectives based on the legal, financial and administrative needs of the collaboration. Sometimes these are requirements from the funding source or the HUG administration. The informal aspects come from shared values, interests and previous collaborations. This formal and informal process is also found in the deliberation process of the collaboration. For example in the Peru collaboration an informal process was taken throughout with no formal contract, terms of reference or methods of communication and reporting. In contrast for the Kyrgyzstan medical education reform project much more structured management and communication processes are in place.

### Shared motivation

Focusing on the interpersonal and relational aspects of the collaboration process different projects within the DTHM portfolio highlight that these factors can impact the other elements of the collaboration. The Mali technical support for diabetes is built on a long-standing collaboration and friendship. Friendship is also an ingredient in the nursing related projects in Tanzania, Togo, and Bosnia-Herzegovina. This leads to mutual trust as the relationship is not only professional, but also personal contributing to mutual understanding in terms of the relationship that has been developed. However, as the relationship goes beyond purely being professional there is a different form of respect of colleagues. For example in both NCD projects in Mali and Peru, open and honest discussions about progress, challenges and next steps could be had from both a professional and personal perspective with a level of frankness that allowed difficult issues to be addressed, for example with demands from donors, issues with publications and involvement of different partners.

Trustworthiness and credibility are both built during previous collaborations, interactions within existing networks or through other partnerships. The trust and credibility of DTHM staff is created through their expertise, which is made visible through publications, participation in different conferences and meetings and being part of different expert groups and networks. For example the DTHM’s involvement in the area of chronic diseases in humanitarian action is built on the strengths that the division has in both of these elements. Through the participation, expertise and work in the area of NCDs and credibility gained in that field and networks the DTHM was able to engage in this new area of activities. All these elements lead to shared commitments to the collaboration as this is built on a mix of professional and personal factors that mean that the success of the collaboration is more to the individuals involved than just something that needs to be successful for donors.

## Capacity for joint collaboration

The partners involved, their institutions as well as any requirements of the donors, determine procedural elements. Being based at a public hospital means that many administrative challenges exist in trying to implement projects abroad, in an institution that is set-up, from an administrative perspective, to deal with delivery of healthcare in Geneva. Different procedural elements exist within the different collaborations with institutional agreements going beyond just the DTHM. In Bosnia-Herzegovina (i.e. in the two entities, each with specific strategies and political governance), where the HUG has been involved for almost 20 years the different elements of this collaboration have been translated into various agreements, conventions and memorandums of understanding. Thanks to this (and only thanks to this), was it possible to embark on such a large and complex project that aims at reforming nursing care in the whole country.

With regards to the different roles of leadership, again staff within the DTHM and their colleagues assume these roles dependent on the aspect of the project and also where the role needs to be performed. For example for the same project DTHM may represent the project in Switzerland, whereas local colleagues assume this role in the country where the project takes place. In Kyrgyzstan the DTHM and local colleagues from an NGO play the role of facilitation for local partners in the Ministry of Health, Medical faculties, Professional Medical Associations and other partners.

Knowledge sharing is challenging across such diverse projects as linguistic and cultural factors play an important role. Materials often need to be translated, if not translated twice, as well as adapted to local contexts. Experience from Kyrgyzstan shows that many documents from Switzerland need to be translated from French to English and then again to Russian. This allows local colleagues to discuss the results of joint projects or technical documents, before using these with other partners. This challenges knowledge diffusion, as this is time consuming and requires more scrutiny. The translation into culturally appropriate materials or approaches is enabled by the experience and expertise of the DTHM and their local colleagues in working in international projects. Management of cultural factors needs to be an integral part of some of the projects, as for example in Bosnia-Herzegovina where there the DTHM is working not only in two different entities, but also in a consortium manner with one Bosnian foundation and two Swiss counterparts. In terms of mechanisms for sharing knowledge, technology such as e-mail, web-based videoconference tools and document sharing software make this process easier, but mechanisms need to be put in place to effectively use these tools.

The main resources that the DTHM and in-country colleagues provide to these collaborations are human resources, with their different experience and expertise. DTHM human resources include ten practicing doctors (including a Professor head of the Division) with six doctors involved in international and research projects and six nurse practitioners with one also involved in development projects and four dedicated project and research staff, including two PhDs in public health and a health economist. In addition the DTHM can call upon other human resources from the HUG and University of Geneva. Many of the financial resources necessary for these projects come from the public sector in Switzerland, either at Federal or Cantonal level. These often cover part of the salary costs of staff within the department, as well as costs for in-country partners. Many of the research sources do not cover substantial costs linked to salaries, either due to their conditions or the amount of resources available. Most of the funding raised for collaborations is done in Switzerland for the benefit of partners. Although the financial contribution to collaborations is minimal from partners, their in-kind contributions in terms of staff time, facilities, networks and knowledge should not be discounted.

## Discussion

The aim of this analysis was two-fold. Firstly, to apply the concept of collaborative governance to international health projects. Its second aim was to highlight the lessons learnt from the DTHM’s experience within this framework. Limitations to this approach are that only part of Emerson et al.’s [17] model was used in the analysis, with the choice of elements seen as most interesting in how collaborations are established and run. Another element included in Emerson et al.’s [17] model are the outputs of the different analyzed projects. The outputs of these different projects could be measured in terms of their achievement of stated goals, management of resources, satisfaction of partners and donors or scientific output. Lasker et al. [10] in their proposed framework for looking at outcomes of collaborations focus on: satisfaction of stakeholders; quality of partnership plans; sustainability of partnership; changes in community programs; policies and practices; and improvements in population health indicators. This overlooks an important aspect, which is the process of collaboration that allows these outcomes to be achieved. Many of the frameworks in Table 2 focus on “Capacity for joint collaboration” and only Huxham et al. [13] discuss the issue of “Members of the collaboration”. Although many models exist a clear deficiency in the literature on international partnerships is the lack of focus on the individuals and their skills and role within the partnership that Emerson et al.’s [17] model addresses. Other limitations are clearly that the analysis was carried out by those directly involved in the different projects and was only carried out from the DTHM’s perspective.

To the authors’ knowledge this is the first time such an analysis has been carried out using collaborative governance to assess global health partnerships. Traditionally collaborative governance in the health related literature has focused on collaboration from an inter-agency [23–25], cross-sectorial [26], public/private [27], inter-institutional [28], inter-disciplinary [29] and inter-professional perspective [30, 31].

Lessons learnt are presented from the “Northern” perspective. However this is an important focus as the concept of partnerships needs to look at how these can be mutually beneficial and therefore further engage “Northern” institutions in seeing the value of such collaborations. The DTHM being based at a publicly funded institution, with as its primary focus the health of the population of Geneva, needs to ensure that its management at the HUG sees the added value of this type of work. One perspective is that projects such as Ebola and NCDs are global health problems. Therefore the “North-south” dichotomy should be ignored as these problems and their solutions will only be addressed by global cooperation [32]. This is linked to partnerships being mutually beneficial as discussed in the literature on international partnerships [5, 7–9, 11]. From the DTHM’s experience, a clear focus on specific areas of activities, where the department and staff can be seen as leaders in the field, is important. This definition of being leaders, is developed through networks, publications, conferences and meetings, where the visibility of the individual and institution can be exposed. Within each described collaboration, each partner “brings something to the table”, without which the partnership might not be possible, e.g. human resources, technical expertise and funding, as well as shared values and interests. With financial resources predominantly coming from the Northern partner, in kind contributions from Southern partners should not be neglected. The management of the partnership resources (e.g. financial and human) needs to be adapted to the local context and partners. This is also how the partnership is defined and managed, with a more formal or informal approach. The overall approach and management of the partnership is closely linked to the friendship that leads to or is the result of the collaboration. This is highlighted by Gaillard [2] as something just as important to ensure success. Another facilitator of the different collaborations, is the effective use of communication technology, to ensure ongoing communication for the pursuit of the project, and needs to be used effectively.

Lessons learnt from the DTHM international partnerships highlights, that often partnerships are thought of as interactions between two partners, but there are other partners involved, e.g. funders and secondary partners. This adds to the complexity of managing these partnerships. Besides this complexity, these other partners bring additional expertise to the overall project. The DTHM in this case, plays a role at the HUG as an expertise broker and link between external partners and additional resources at the HUG and University of Geneva. This additional technical expertise is important, as it is part of the credibility of the DTHM and its staff. In addition to this, technical know-how is also the practical experience that the DTHM can bring to its partnerships. This experience and the skills mix within the DTHM allow for innovative context specific responses to be developed. Networks also play an important role in both increasing the visibility and credibility of the DTHM and its staff and serving as platforms for the development of new collaborations.

As discussed by Leather et al. [5] international partnerships can also be beneficial to the “Northern” partner, due to changes in the diversity of the patient population and the globalization of health issues. For example the expertise gained in the South can be useful in the management of health problems for migrant populations. They can increase reputation and visibility of the organization beyond its geographical boundaries and traditional areas of activity [11, 33]. Also highlighted in the literature are the advantages for the individuals involved, in terms of their personal and professional development [5, 11, 33], as well as new skills or new ways of applying existing skills [5, 10, 11, 14, 33]. In the UK, barriers to the scaling up of North-South links, often include costs to the National Health Service for staff time away from the UK, and a lack of volunteers in a position to spend extended periods of time overseas.

## Conclusion

Using collaborative governance to analyze international collaborations, allows for an interesting approach, as this model usually focuses on the complexity of managing partnerships across different types of actors with varying interests, which is a key element of international partnerships. It also helps in highlighting how these partnerships might be important for the Northern partner. As stated by McKee and Healy [34] hospitals must adapt to changes in society, technology and health needs. Partnerships allow this process of adaptation to occur as staff at the Northern institutions is challenged in their way of doing things through these partnerships. One skill discussed is creativity in that partnering with other countries, institutions and colleagues with different backgrounds which enables exposure to different views, approaches and skills [10, 11, 14]. Job satisfaction may also be another benefit of organizations proposing international collaborations [11, 33]. Syed et al. [11] add that partnerships may also lead to better job satisfaction and also state that there are many intangible benefits of partnerships. Although the role of the HUG is the health of the population of Geneva, the forces of globalization are such that the boundaries of the HUG need to go beyond the geographical boundaries of the population it serves. The experience gained by DTHM staff through this work not only enables them to develop a unique set of skills in working in international partnerships, but also strengthens their role as clinicians, managers, researchers and teachers for the benefit of the HUG as hospital, institution and academic center. This is helped by the leadership of the HUG [35], inclusion of the HUG’s humanitarian role in its latest strategic plan [36], innovative support mechanisms from the special humanitarian fund as well as the overall role of the HUG as a teaching hospital and center of excellence.

## Abbreviations

ABHRS: alcohol-based hand rub solution
BPKIHS: B.P. Koirala Institute of Health Sciences
DTHM: Division of Tropical and Humanitarian Medicine
HUG: Geneva University Hospitals
KFPE: Swiss Commission for Research Partnerships with Developing Countries
MSF: Médecins sans Frontières
NCD: noncommunicable disease
NTD: neglected tropical disease
SDC: Swiss Agency for Development and Cooperation
UK: United Kingdom
WHO: World Health Organization
